# Asthma

**Published:** 1885-09

**Authors:** 


### Asthma

Is an incurable disease by human agencies. An attack can be modified or shortened, and this is all that the thousand and one vaunted remedies for the "cure" of asthma can do; they alleviate or remove for the time, nothing more. Sometimes the disease lies dormant for months or years, only to reappear in some change of life, or some more terrible form of human affliction. In some cases it disappears in childhood, to show itself again after forty years. If it disappears at the "change of life," it may not be heard of again, but that life will seldom reach three-score and ten.

It is very certain persons troubled with asthma may be exempt from it for a succession of years, and even for life, by removing to a different atmosphere or a different climate; hence, instead of losing time in the attempt to "cure" asthma or of being satisfied with shortening or curing merely an attack of it, it would be a wiser course to change localities or climates Standard medical works give interesting cases in which exemption more or less permanent is secured by moving from the city to the country, from a level to a hilly locality, from the seaside to inland, from Maine to Florida, from Cape Cod to California, and the reverse of all these; that is, an entire change of air does sometimes exempt from asthmatic attacks.

When such changes are impracticable from any cause, the next best thing is not to have asthma at all. It is exceedingly inconvenient to be blowing like a porpoise, to feel as if fifty thousand feather pillows were piled upon your face, to have the sensation that you would certainly die if you stopped breathing long enough to say "Yes" or "No;" and yet such are the sensations during an attack of asthma. And what is worse, these worse than inquisitional tortures usually come on towards daylight, the very time when sleep is most delicious; you are obliged to sit up in bed, and with your head leaning forward on your drawn-up knees you feel, until the longed-for daylight comes, and sometimes for hours later, as if every breath would be your last, and mortal agony is depicted in every lineament of the face.

But there is, as the poet says, "comfort in brooks and stones, in everything." Asthmatics never die; they can whistle at consumption, and bronchitis, and all such tough customers; that is, asthmatics generally live to old age, unless this form of the disease disappears; asthma itself is antagonistic of consumption. In consumption, you can't get air enough in to support life, because there are not lungs enough to receive it; in asthma, you can't get the air out, hence, remaining in, it gets warmer, and distends the lungs by its increasing rarefaction, and thus develops them.

Asthma is almost always the result of a bad cold attacking an asthmatic constitution. This cold forms phlegm in the branches of the windpipe,—a tough, sticky phlegm, which adheres to the insides of these hollow branches of the windpipe; and as the air goes into these branches with more force than it comes out, it has the effect to carry the phlegm before it, downwards, in the shape of a plug, into a narrowing orifice, every breath that is drawn. But it is with asthma as in other diseases; when they reach their worst, that is, their "crisis," they begin to get better. It is not necessary to say how, just here, but the suggestion is repeated, that it would save a great deal of suffering and trouble if persons would only not have asthma; and all that one has to do is simply to never take a cold; and certainly a man need not take a cold once in a year or five or ten years. All that he has to do is to dress abundantly warm to avoid getting chilled, and to cool off slowly after being over-heated; that is, after being warmer than is natural. Such is the true philosophy of common asthma.

